# Rapid and selective surface functionalization of the membrane for high efficiency oil-water separation via an atmospheric pressure plasma process

**DOI:** 10.1038/s41598-017-15713-x

**Published:** 2017-11-10

**Authors:** Yong Sung You, Seongchan Kang, Rodolphe Mauchauffé, Se Youn Moon

**Affiliations:** 1 Department of Quantum System Engineering, Chonbuk National University, 567 Baekje-daero, Deokjin-gu, Jeonju-si, Jeollabuk-do, 54896 Republic of Korea; 2 Department of Applied Plasma Engineering, Chonbuk National University, 567 Baekje-daero, Deokjin-gu, Jeonju-si, Jeollabuk-do, 54896 Republic of Korea

## Abstract

Oil-water separation is a worldwide challenge because of the increasing production of industrial oily wastewater and frequent oil spills. The growing environmental and economic demands emphasize the need to develop effective solutions to separate oil and water. Recently, oil-water separation methods were developed by tuning the wettability of membranes via surface functionalization. However, the industrialization of such methods remains challenging due to the easy-fouling, high cost and complex fabrication. Herein, a simple and rapid pathway to separate oil from oil-water mixtures is reported using plasma surface functionalization in an open-air environment. The fine tuning and study of the plasma process parameters enables the selective functionalization of each side of the membranes which led respectively to a superhydrophobic-superoleophilic and superhydrophobic-oleophobic sides. The successful separation, without any external force, of a 50 mL oil-water solution in 6 minutes was achieved. This work paves the way for an efficient, low cost and easily upscalable method for oil-water separation due to the high versatility of the atmospheric pressure plasma processes.

## Introduction

Oil-water separation is one of the major global challenges because of the increasing industrial oily wastewater production in the petrochemical, textile, and food industries, for example^1–3^. The exploration and production of oil generates a large volume of wastewater, known as “produced water”, and requires efficient de-oiling methods to treat the water before its reutilization^3^. Frequent oil spills, such as the recent release of oil into the Gulf of Mexico as a consequence of the explosion of the “Deepwater Horizon” oilrig in 2010, are also urgent global environmental problems^4^. Such environmental and economic concerns clearly demonstrate a strong need to develop efficient oil-water separation methods. Currently, techniques including gravity separation, filtration, centrifugation, flotation or electrochemical methods are commonly used; however, in addition to their relatively low efficiency, they are associated with high operational costs and require a long processing time^5–7^.

Recently, to overcome such drawbacks, a highly efficient alternative pathway was reported, consisting of the functionalization of the membrane surfaces to provide selective water/oil wettability surface properties^2,7–14^. Indeed, the functionalization of the membrane enables water or oil to be selectively repelled or allowed to pass through it^7–14^. Various approaches are reported in the literature for the functionalization of membranes, including polymer-grafting^7–10^, electrospinning^11^, self-assembly^12,15^, selective etching of the surface^13,16^, nanotube growth^14^, and lithography^17^. Those methods enable the tuning of the surfaces’ wettability through the control of their surface chemistry, i.e. surface free energy and surface morphology^14–18^. However, for industrial implementation and large-scale production, some limitations remain, such as the high cost, complex fabrication procedures, low stability and flexibility, and poor selectivity and reusability^3,9,18^.

However, alternative surface functionalization methods are available, such as the chemical vapor deposition (CVD) processes^13,18–20^. They are particularly suitable for industrial upscaling as they allow a one-step deposition that avoids the use of solvent and drastically reduces the generated wastes. Among the CVD processes, some are performed at a low pressure such as the initiated CVD or aerosol-assisted CVD, but still have similar limitations^19,20^. Therefore, atmospheric pressure processes, which avoid the use of vacuum equipment with high operational costs and is more easily implemented in an existing in-line process, was then developed. While atmospheric pressure CVD presents a high growth rate, it still requires substrate heating; thus, it is limited to non-heat sensitive materials. This limitation is the reason the emerging atmospheric pressure plasma technology is the method of choice for surface functionalization. Atmospheric pressure plasma processes are low cost, allow the treatment of heat-sensitive materials due to their low temperature plasma, can be performed in open-air, offer the possibility to treat large areas, and enable the fast modification of surfaces with readily tunable chemistry and morphology^21–24^.

In this report, the rapid fabrication of mesh membranes that have both a superhydrophobic-superoleophilic side and superhydrophobic-oleophobic side for effective oil-water separation via atmospheric pressure plasma treatment is detailed for the first time. In the first part of the process, the fine control of the plasma parameters, and more precisely the feed gas composition, enables the control of the surface wettability. The formed coatings and wettability changes from the origin were studied through the plasma discharges diagnostic via Optical Emission Spectroscopy (OES), surfaces morphology observation by Scanning Electron Microscopy (SEM), and the surfaces chemistry analysis via X-ray Photoelectron Spectroscopy (XPS). In the second part of the process, the oil-water separation efficiency study of coated mesh membranes was conducted. Both the separation capability and mesh membrane reusability were assessed.

## Results and Discussion

### Surface wettability control

**Figure 1:**
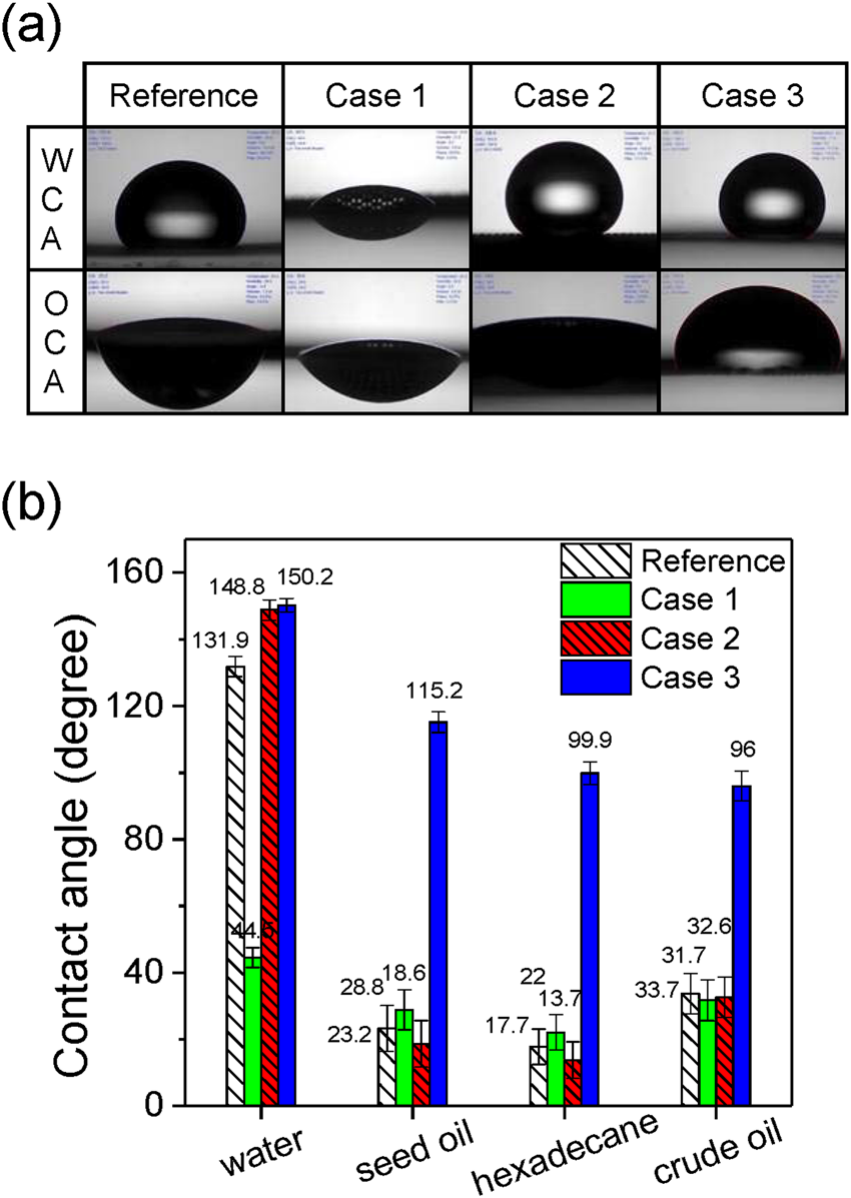
(**a**) Static contact angles of water (WCA) and oil (OCA) on an untreated membrane (Reference) and single side plasma treated membranes for cases 1, 2 and 3. (**b**) Contact angles measurements of water and the various types of oil on the reference and the single-side plasma treated membranes in air.

Plasma surface modification of a stainless steel mesh membrane has the benefits of being low cost and presenting good mechanical and chemical stability. It was conducted in order to form an oil-water separating membrane using a cylindrical atmospheric pressure plasma source (Supplementary Figure S1). As reported in a previous study, the atmospheric pressure plasma method easily enables the formation of superhydrophilic, hydrophobic or superhydrophobic surfaces^21–24^. Based on these findings, via the plasma parameters control in Table 1, selective water/oil wettability properties were conferred to metal mesh membranes (Fig. 1a and b). The reference mesh membrane surface (mesh number 120, pore size ~124.5 μm) exhibited an intrinsic hydrophobic-oleophilic property according to water contact angle measurements (WCA) and oil contact angle (OCA) measurements using various oils (Fig. 1b). After treating a single side of the metal membrane surface with a pure helium discharge (Case 1), which is known to lead to the surface formation of hydrophilic groups (i.e. -O and –OH), the surface became hydrophilic and oleophilic. The fine control of the injected CH_4_ and C_4_F_8_ flow into the helium discharge enabled the selective control of the surface’s water/oil wettability. By adding both CH_4_ and C_4_F_8_ gases to the helium plasma (Case 2), the plasma treated mesh surface displayed a superhydrophobic-superoleophilic property. Interestingly, when introducing only C_4_F_8_ into the helium discharge (Case 3), the surface remained superhydrophobic but the OCA increased up to 115° for the seed oil, which indicated an oleophobic property. Since the surface wettability is governed both by the surface morphology and surface chemical composition^14–25^, physical and chemical investigations were conducted to highlight the different mechanisms leading to the oleophilic (Case 2) and oleophobic (Case 3) properties.

**Table 1:** Atmospheric pressure plasma treatment conditions.

Condition	RF power [W]	Time [sec]	Distance [mm]	He [lpm]	CH_4_ [sccm]	C_4_F_8_ [sccm]
Reference			Untreated sample			
Case 1	180	120	1	4	0	0
Case 2				4	40	10
Case 3				4	0	10

**Figure 2:**
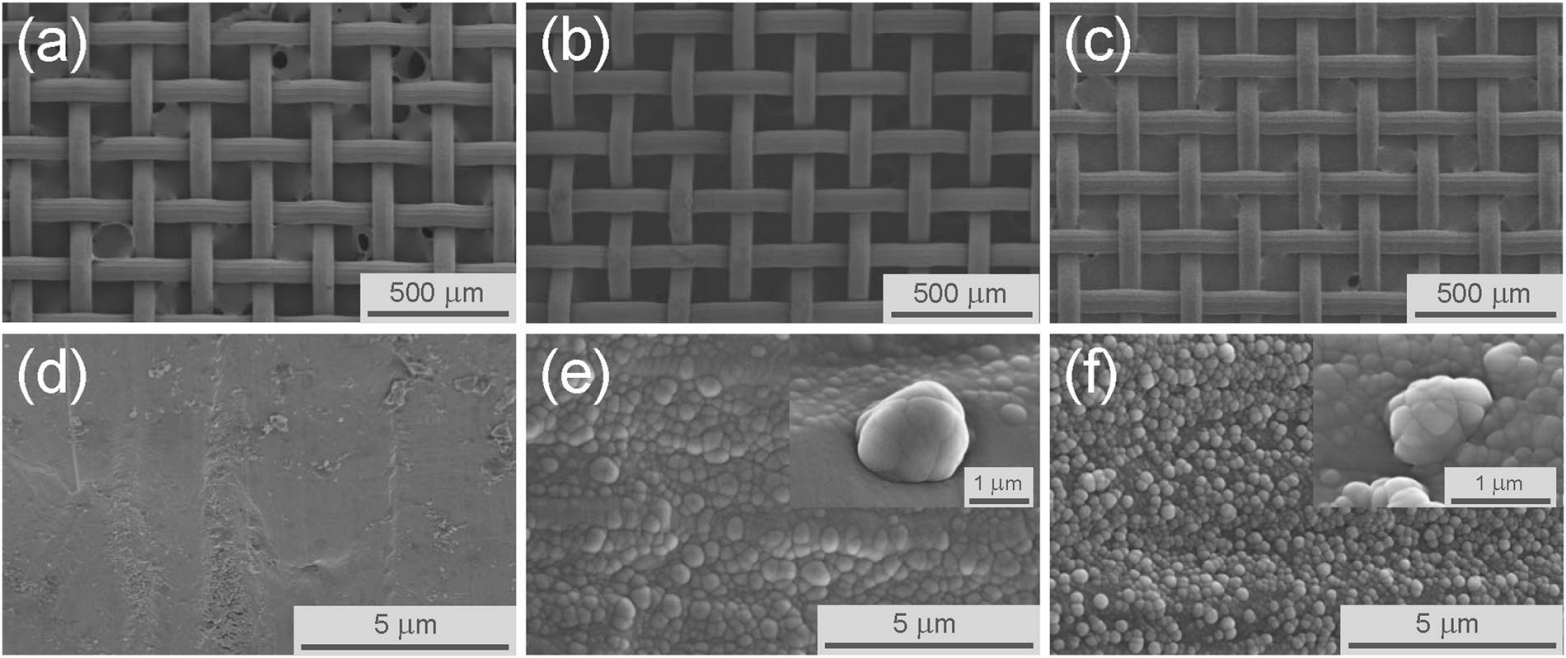
SEM images of the reference stainless steel membrane (**a**) and of the plasma treated membranes with case 2 (**b**) and case 3 (**c**). Magnified view of the knitted wire surface of the untreated membrane (**d**) and of the plasma treated surfaces with case 2 (**e**) and case 3 (**f**).

The reference mesh membrane surface, which was observed by SEM (Fig. 2a and d), appeared to be smooth. After atmospheric pressure plasma treatment using the conditions of cases 2 and 3 (Fig. 2b and c), the macroscopic morphologies of the membranes appeared similar to the reference membrane (Fig. 2a). However, as displayed in Fig. 2e and f, at higher magnification, a thin-film with a hierarchical micro-nano structure was noticeable in both cases. Nano-aggregates with sizes ranging from several tens to several hundreds of nanometers were observed (insets of Fig. 2e and f). Since the lack of significant morphological differences between cases 2 and 3 did not explain the different observed oil contact angles, surface chemistry analyses via XPS were carried out.

**Figure 3:**
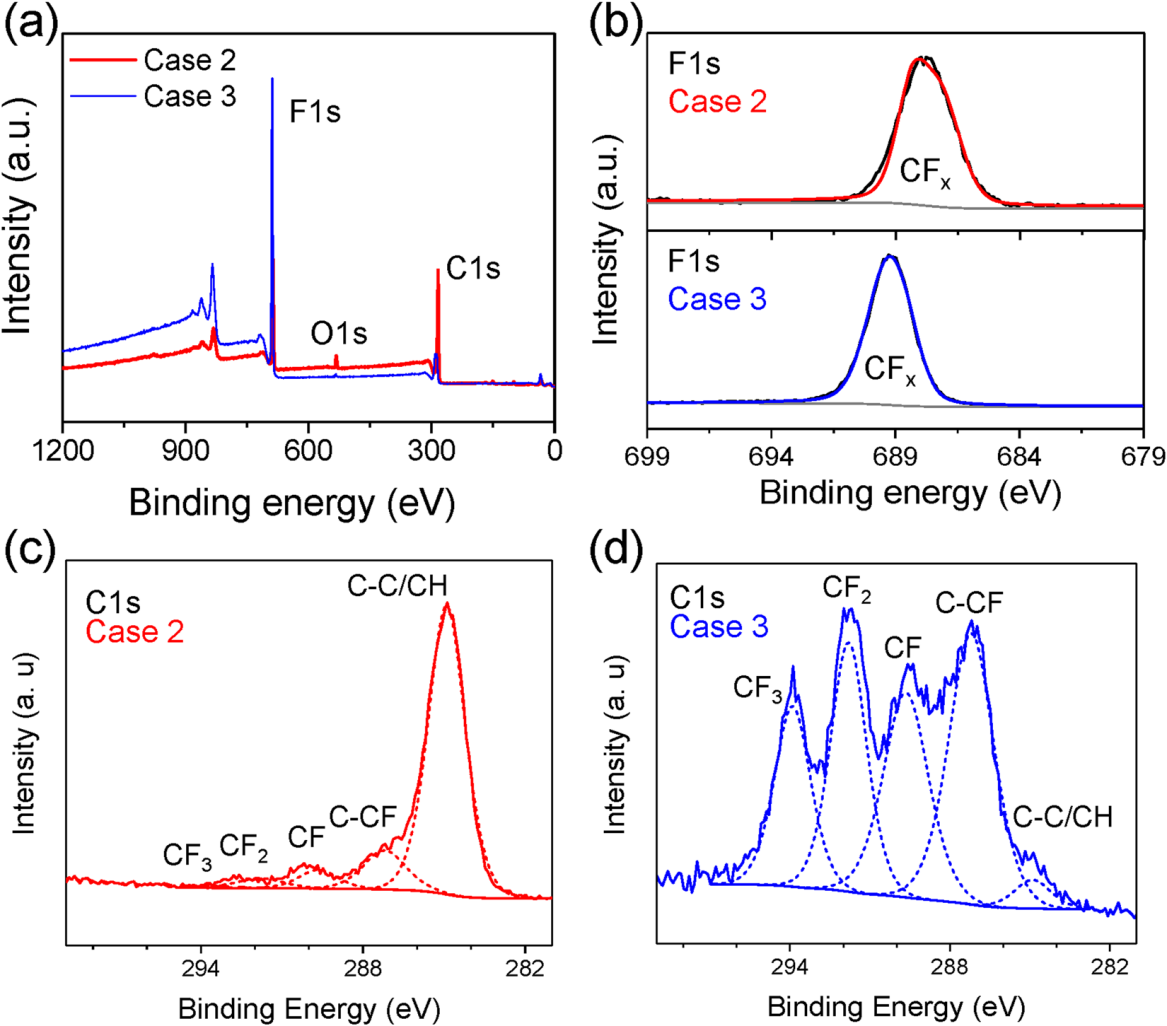
(**a**) XPS survey spectra of plasma-treated membranes with cases 2 and 3. (**b**) High-resolution F1s spectra for case 2 (top) and case 3 (bottom). C1s core level fitting for case 2 (**c**) and case 3 (**d**).

As reported in Fig. 3a and Table 2, whereas the introduction of C_4_F_8_ into the helium discharge (Case 3) led to coatings with a high fluorine surface concentration, the introduction of CH_4_ in concert with C_4_F_8_ (Case 2) drastically increased the carbon content at the surface of the deposited thin films. The C1s curve fitting (Fig. 3c and Table 3) suggested that in case 2, carbon is primarily involved in the C-H or C-C (284.9 eV) bonds. For case 3, however, (Fig. 3d and Table 3) the high contributions of C-CF (287.2 eV), CF (289.7 eV), CF_2_ (291.8 eV) and CF_3_ (294 eV) demonstrated that carbon primarily bonds to fluorine^23^. The F1s peak (Fig. 3b) in case 3 clearly suggested the major presence of covalent bonds between carbon and fluorine, with the presence of the high binding energy CF_x_ species (around 689 eV); while case 2 exhibited a lower energy CF_x_ species (around 687 eV) that was likely due to partly semi-ionic bonds formation and a lower fluorine environment^26^.

**Table 2:** XPS chemical surface quantification for cases 2 and 3.

Condition	C (at.%)	O (at.%)	F (at.%)
Case 2	73.7	3.7	22.6
Case 3	43.1	1.1	55.8

**Table 3:** XPS-based elemental compositions of C1s for cases 2 and 3.

Condition	Assignment	Position [eV]	Relative area [%]
Case 2	C-C/C-H	284.9	78
	C-CF	287.2	12
	CF	289.7	6
	CF_2_	291.8	3
	CF_3_	294.0	1
Case 3	C-C/C-H	284.9	3
	C-CF	287.2	32
	CF	289.7	24
	CF_2_	291.8	24
	CF_3_	294.0	17

These distinctive surface compositions and wettability were likely influenced by the different gas phase chemical elements present in a plasma state. The optical emission spectrum of a pure helium plasma (Case 1) demonstrated that the helium atomic emission lines were dominant with a strong OH (A^2^Σ^+^ − X^2^Π) molecular band and oxygen atomic lines (777 and 844 nm) due to air impurities (Supplementary Figure S2a and b). They were responsible for the hydrophilicity of the surface depicted in Fig. 1. Furthermore, given the plasma effects on the electron impact, the introduced CH_4_ and C_4_F_8_ gas could be dissociated into CH_x_ and CF_x_, respectively, and easily deposited onto the surface. Indeed, the optical emission spectra discharges of cases 2 and 3 (Supplementary Figure S2b and c) clearly demonstrated the presence of CH (A^2^Δ − X^2^Π) and CF_2_ (^1^B_1 _− ^1^A_1_) molecular bands, which aligned with the XPS surface analyses.

We can then conclude according to the similar morphologies between cases 2 and 3 that the different oil wettability behaviors depicted in Fig. 1 primarily resulted from the surface chemical compositions. Indeed, the oleophilic behavior of the case 2 surface can be explained because of the presence of CH_x_ groups on the top surface. Such groups, which were also present in the various oils that were used, led to similar intermolecular interactions between the surface and liquid at the interface that resulted in high surface work of adhesion, i.e. oleophilic surface, according to the Young-Dupre equation^27^. In case 3, however, the higher content of the fluorine-containing species over the surface, led to lower work of adhesion between the oil and coating and thus exhibited a larger oil contact angle.

### Oil-water separation

**Figure 4:**
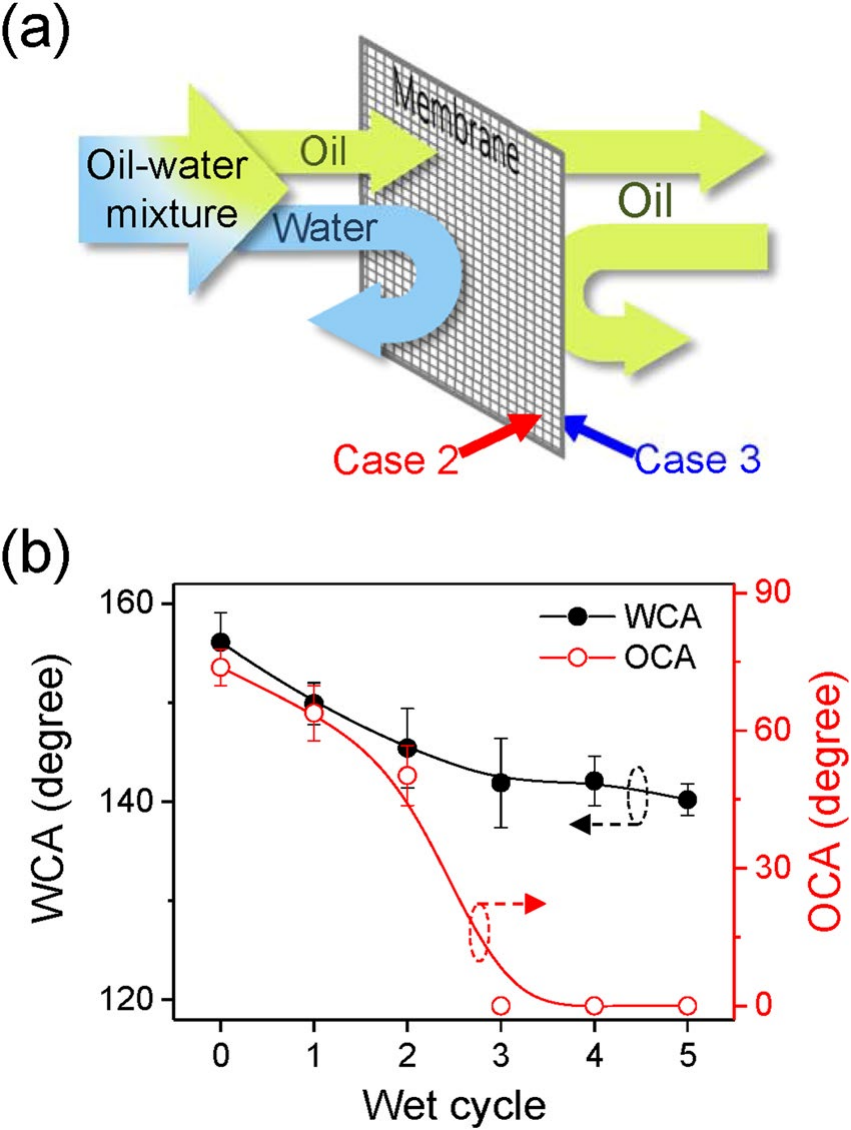
(**a**) Scheme of the oil-water separation system principle. (**b**) WCA (dots) and OCA (circles) of the selectively functionalized membrane surfaces with cases 2 and 3 on each side. The contact angles were measured on the front side of the mesh treated with the case 2 plasma condition, and the back side of the mesh was treated with case 3.

In order to design an efficient oil-water separation system, the stability of both WCA and OCA after water dipping was an important parameter. The stability of the surfaces’ wetting properties was then studied following both WCA and OCA after dipping cycles (referred to here as ‘wet cycle’) that consisted of the full dipping of the membranes for 2 minutes in water (Supplementary Figure S3). The reference mesh membrane, which was not treated and exhibited originally hydrophobic and oleophilic properties, became superhydrophilic and superoleophilic after only one wet cycle, regardless of the mesh number (Supplementary Figure S3a and b). Intuitively, a mesh membrane that was coated only on one side with the case 2 parameters and presented superhydrophobic and oleophilic properties, appeared to be a good candidate for the separation of oil from an oil-water solution; however upon immersion in water, the WCA decreased to 0° after three wet cycles, which made case 2 unusable for oil-water separation (Supplementary Figure S3c and d). This decrease of WCA was then believed to be partly due to the effect of the untreated opposite side of the mesh membrane. Indeed, since the mesh membranes that were used had large pores, water may have slightly penetrated the pores and wetting was likely to be affected by the back side properties at the interface. It is worth noticing that the case 3 coating, when deposited onto a single side of the membrane, had a better stability with WCA and OCA values of 95° and 38.5°, respectively, for 3 wet cycles. The difference between the two cases may be partially due to the contact angle hysteresis which was smaller in case 3 (Supplementary Figure S4). To form an efficient oil-water separation membrane, as shown in Fig. 4a, we then suggested the functionalization of both sides, with case 2 on the front side and case 3 on the back side. As depicted in Fig. 4b, the wet cycle influence on the WCA and OCA values for the front side that previously exhibited unstable wettability upon immersion. The WCA and OCA values were as high as 156° and 75°, respectively. Compared to the substantial decrease in WCA that was reported previously in Figure S3 for case 2 on a single side deposited membrane, in this study, after three wet cycles, the WCA of the double side deposited membrane displayed a nearly constant WCA value of about 140°. The observed differences of wetting behavior compared to the single-side coated case are likely to be due to the influence of the back side properties as treated by case 3. When only one side of the mesh was treated, water residues were trapped in the membrane pores due to the untreated back side after the wet cycle test (Supplementary Figure S5a and b). However, since both sides were modified into the superhydrophobic surfaces using cases 2 and 3, no water residues were observed for the double-side treatment case (Supplementary Figure S5c). Therefore, the WCA of case 2 after a wet cycle test was restored to its initial value by including a dry cycle in open air without any changes in the chemical composition and physical morphology (Supplementary Figure S5d). It was also notable that the OCA was decreasing and approaching 0°. The increased stability of the WCA and the low OCA conferred via the combination of cases 2 and 3 on each side of the membrane was promising for the use of such modified membranes for oil-water separation.

**Figure 5:**
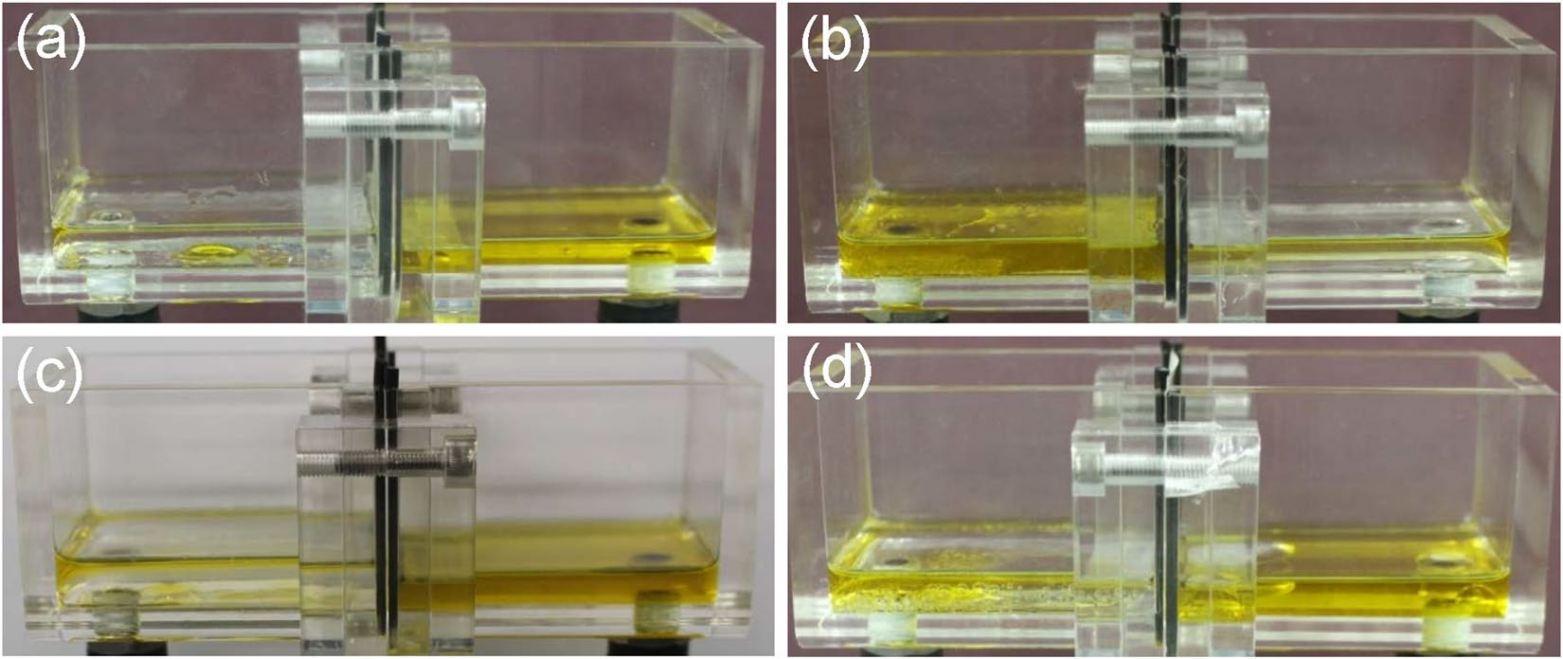
Oil-water separation test using a (**a**) reference membrane, (**b**) pre-wetted (one wet cycle) reference membrane, (**c**) plasma treated membrane with case 2, and (**d**) pre-wetted (one wet cycle) plasma treated membrane with case 2. The 50 mL (1:1 vol.%) oil-water mixture was poured into the left bath and the mesh membrane was placed between the left and right bath.

Therefore, we investigated the oil separation capability of the plasma treated mesh membranes using two baths separated by a mesh membrane as depicted in Fig. 5. To minimize the effect of the pressure applied by the liquid’s own weight, the two baths were placed horizontally. A seed oil-water mixture (1:1 vol. %, total volume 50 mL) was poured into the left bath. The separation test was performed until no changes were noticeable with further separation time. Figure 5a and c display the final oil-water separation results using a reference membrane and a plasma treated mesh membrane with case 2 on a single side, respectively. Owing to their superhydrophobic/oleophilic property, oil quickly permeated through the mesh while water was retained in the left bath. Since the baths were horizontally positioned, total separation was not possible even after a very long separation time, which justified the residual oil that was present in the left bath after the separation test. Moreover, as seen in Fig. 5b and d, when the test was consecutively performed using the same mesh membranes after a simple replacement of the previously separated oil/water solution, the oil-water separation was not reproducible. This behavior was in accordance with the results reported in Supplementary Figure S3. Since both sides of the mesh membrane were previously wetted by water and/or oil, a change in the superhydrophobic and oleophobic properties occurred. Therefore, we assessed the oil-water separation performance of a membrane selectively modified on both sides with cases 2 and 3.

**Figure 6:**
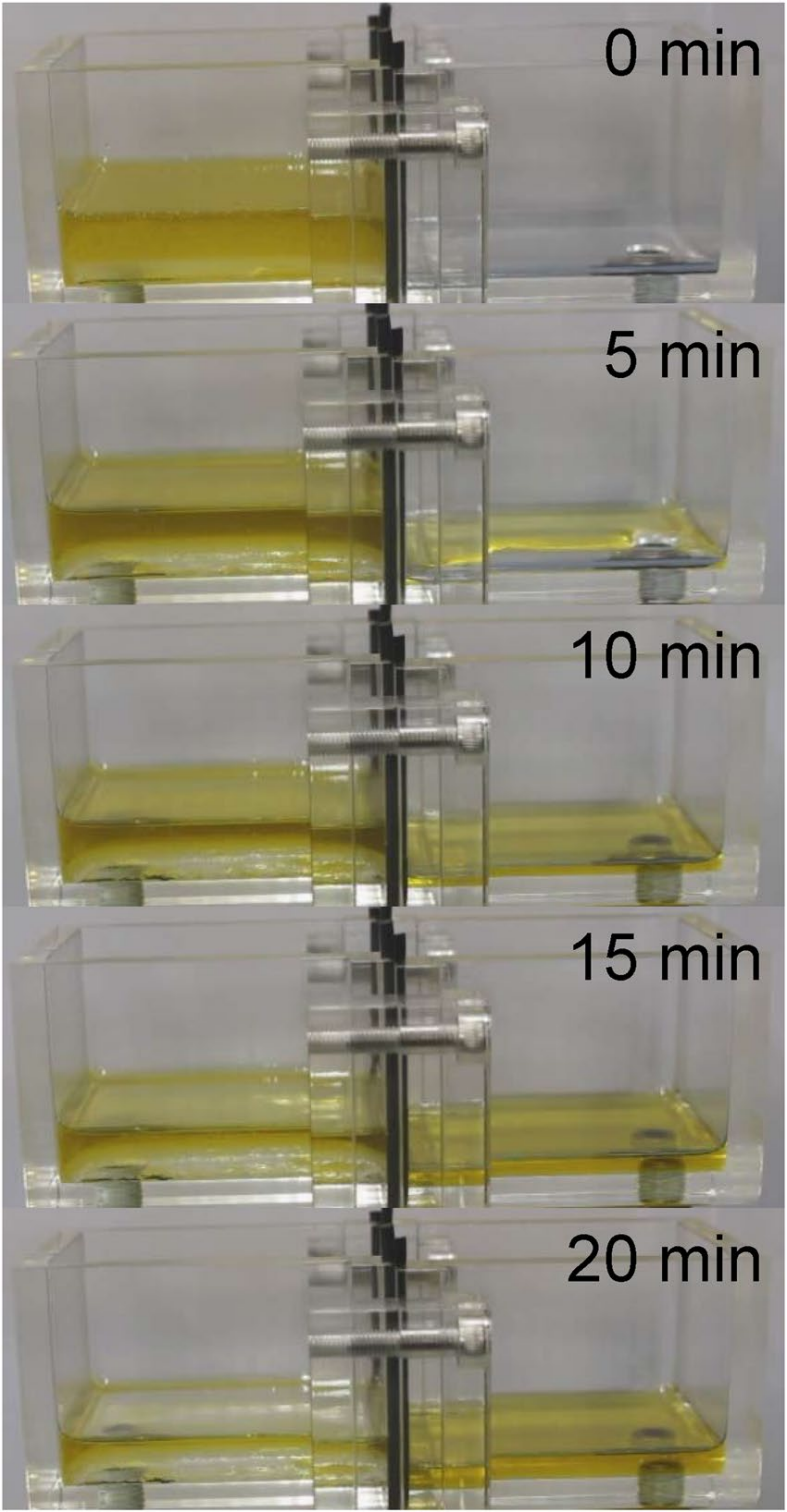
Pictures of the oil-water separation experiment after separation times from 0 to 20 minutes. The plasma membrane, left (front) and right (back) sides, were treated with cases 2 and 3, respectively.

**Figure 7:**
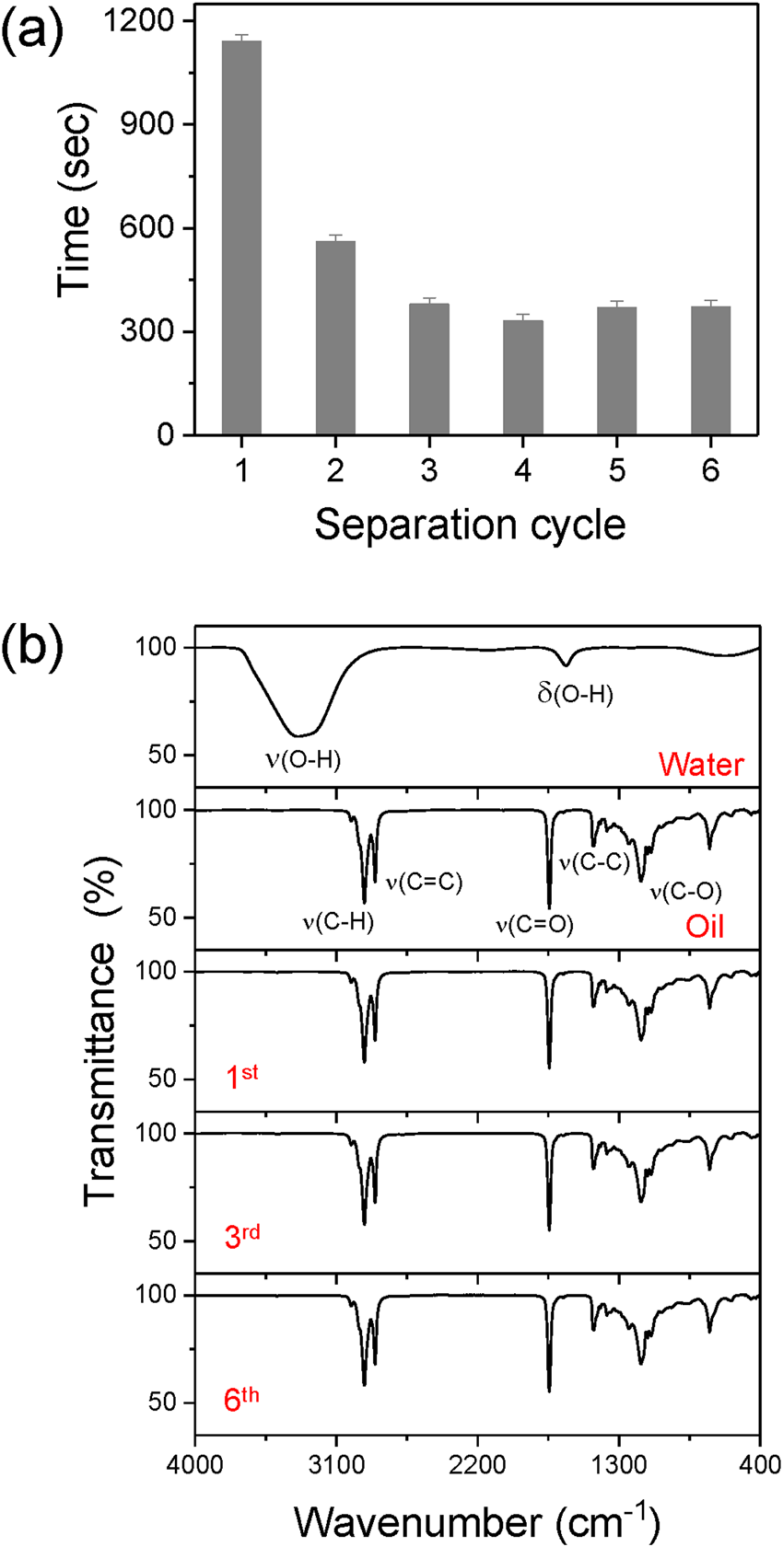
(**a**) Time to separate 88% (22 mL) of the initial oil (25 mL) mixed with water for 1 to 6 successive separation tests. (**b**) The FTIR spectra of water, seed oil, and separated seed oil for the 1^st^, 3^rd^, and 6^th^ tests.

Figure 6 shows the pictures of the oil-water separation experiment at various times with both sides of a plasma treated membrane. We noticed that only oil seemed to progressively permeate through the membrane and appeared to be clearly separated from the oil-water mixture after about 20 minutes. An as-prepared mesh membrane requires approximately 20 minutes to separate 88% of the oil from the mixture. As reported previously, because of the static horizontal baths’ position without external pressure, 100% separation yield could not be reached and was limited to approximately 88%. Since the selectively functionalized membrane exhibited a better superhydrophobic/superoleophilic property after few wet cycles (Fig. 4b), we performed several separation cycles and monitored the duration to fill the 22 mL volume of the right bath (separation yield 88%) by oil separation. A clear improvement was observed at only 6 minutes, which was what was required to separate the same volume of oil after three separation cycles (Fig. 7a and Supplementary Movie 1). The purity of the separated oil was assessed by a FTIR analysis (Fig. 7b). The FTIR spectra was obtained for water, seed oil and separated collected oil after one, three and six separation cycles. No water related peaks, such as the O-H peak, were observed in the separated oil FTIR spectra which suggested a very high separation efficiency of the selectively functionalized mesh.

The efficient and reproducible oil separation from an oil-water mixture combined with an easily upscalable atmospheric pressure plasma treatment process makes the approach described herein particularly suitable for industrial use. Such coatings that present superhydrophobic-oleophilic and/or superhydrophobic-oleophobic properties, however, are not only limited to oil-water separation. Indeed, amphiphobic surfaces that possess both water and oil repellency properties, i.e. case 3, may be used in applications such as anti-bio fouling surfaces, stain-free material, spill-resistant protective wear, self-cleaning surfaces, and drag reduction material^10,11,21,28,29^. Therefore, in order to develop artificial amphiphobic surfaces currently, efficient but complex techniques have been extensively studied; in this study, such surfaces (Fig. 1b) were simply realized with a 2–minute process using atmospheric pressure plasma treatment by simply adding C_4_F_8_ gas (case 3)^10,11,21,25,26,28–31^. The cold plasma temperature and the various available designs of the plasma sources allowed deposit coatings on a large range of substrates and geometries with a large choice of precursors^32^. It is possible to combine plasma sources to deposit via the same process on both sides of a substrate simultaneously, which exhibited the high versatility of the atmospheric pressure plasma processes and their potential for industrial use (e.g., for the method developed in this study).

## Conclusion

In conclusion, an efficient oil-water separation process was developed via the rapid functionalization of both sides of a membrane which formed a superhydrophobic-superoleophilic side and a superhydrophobic/oleophobic side by atmospheric pressure plasma treatment. Through the simple tuning of the plasma gas composition, a 2-minute plasma treatment enabled the control of the surface oil/water wettability. The XPS analyses clearly highlighted the importance of the surface chemistry; indeed, the surface presence of various concentration ratios of CH_x_ and CF_x_ groups was shown to be responsible for the observed surfaces’ wettability properties. To enhance the separation capacity and reusability, both sides of the mesh were selectively functionalized. Approximately 88% of the oils in the oil-water mixture were successfully separated in 6 minutes without any external forces, and no water was detected in the collected oil according to the FTIR analysis. Since atmospheric pressure plasma processes are easily upscalable and fast, and require low running cost, we expect this method to be widely adopted for the mass production of oil-removing membranes.

## Methods

### Sample preparation

Five different types of stainless steel mesh membranes that had a mesh number of 120, 100, 80, 60 and 50, were used. The mesh membranes (25 mm × 70 mm) were cleaned with isopropyl alcohol (IPA) and deionized (DI) water and dried under compressed air prior to plasma processing.

### Atmospheric pressure plasma deposition

The surface treatment was conducted using a cylindrical atmospheric pressure plasma source (Supplementary Figure S1). A 180 W 13.56 MHz RF discharge was ignited using a RF power supply (PTS PG0.313) through an impedance matcher (PTS PM0.313), which minimized the reflected power to under 1%. Both the outer surface of the plasma source body and the bottom moving table were electrically grounded. A stable homogeneous plasma discharge was obtained using 4 liters per minute (lpm) of Helium. The C_4_F_8_ and CH_4_ gases were selectively added to the helium plasma using two mass flow controllers (MFC, AtoVac AFC500). The plasma discharge was analyzed by optical emission spectroscopy using a spectrometer (SCT-320 Princeton Instruments) equipped with a charge coupled device (CCD, PIXIS400B Princeton Instrument). The substrate was fixed on a moving table and the speed was set to 10 mm/s to treat the whole area of the mesh membrane for 120 seconds.

### Water Contact Angle (WCA) and Oil Contact Angle (OCA) measurement

Deionized water (DI) was used for the WCA tests and seed oil (Sempio Foods Company), and hexadecane (Alfa Aesar) and crude oil (SK lubricants Co., LTD) were used to evaluate the oil contact angle (OCA). Static contact angles were measured promptly using a contact angle measuring instrument (SmartDrop, Femtofab Ltd., Korea). For the measurement of WCA, the water droplet (12 μL) was automatically injected through a needle from a deionized water tank at 25 °C. The static oil contact angle (OCA) was measured using the same equipment with direct oil droppings (20 μL) by a pipette (Eppendorf Research plus). The tilting method was employed to measure both the advancing and receding water contact angles with the same goniometer. After dispensing a 13 μL droplet, the plate was tilted from 0° to 90° at a rate of 0.11°/s. Hysteresis, which was defined as the difference between the advancing and receding angles, was measured at certain tilting angles of 20° and 30°. In order to study the influence of previous immersion in water on the resulting OCA and WCA, the mesh membranes were completely immersed in DI water for 2 minutes (called herein a ‘wet cycle’) before the contact angle measurements.

### Surface characterization

The treated mesh membranes; surfaces were examined using a Field Emission Scanning Electron Microscope (FE-SEM, Hitachi SU-70). X-ray photoelectron spectroscopy (XPS) analyses were conducted on samples using a Thermo Fisher Scientific K-Alpha instrument that had a monochromatic Al Kα X-ray source (1486.6 eV). The C1s core level was fitted with five components for cases 2 and 3: C-C/CH (284.9 eV), C-CF (287.2 eV), CF (289.7 eV), CF_2_ (291.8 eV) and CF3 (294 eV)^23,26^. For clarity, a single CF_x_ contribution, which was comprised of the various CF bonds, was reported for the F1s peaks. For all of the XPS spectra, a Shirley background was assumed and core levels were fitted using a combination of Gaussian and Lorentzian distributions. The FT-IR analyses of the separated solutions were conducted on a PerkinElmer FT-IR/NIR Spectrometer Frontier apparatus that operated while in the attenuated total reflectance (ATR) mode. All spectra were recorded in the 4000 to 400 cm^−1^ range with a 4 cm^−1^ resolution.

### Oil-water separation test

The oil-water separation tests were performed using a reactor composed of two baths separated by the mesh membrane as depicted in Fig. 5. A 50 mL oil-water mixture that was composed of 25 mL of water and 25 mL of seed oil was vortexed and spilled in the left-side bath of the reactor to continue the oil-water separation. The separation was performed with their own liquid weight pressure without additional external pressure. The filtered oil was then analyzed via a FTIR to detect any water presence.

## Electronic supplementary material

Movie clip

Supplementary information
