# Supplementary material for: Rapid and selective surface functionalization of the membrane for high efficiency oil-water separation via an atmospheric pressure plasma process
Source: Sci Rep. 2017 Nov 10;7:15345. doi: 10.1038/s41598-017-15713-x (PMC5681647; doi:10.1038/s41598-017-15713-x)
Supplement: Supplementary file 2 — Supplementary information [file 41598_2017_15713_MOESM2_ESM.pdf]

Supporting Information for

Rapid and selective surface functionalization of the membrane for high efficiency oil-water separation via an atmospheric pressure plasma process

Yong Sung You,[1] *Seongchan Kang,*[2] *Rodolphe Mauchauffé,*[1] *and Se Youn Moon**[,1,2]

[1]Department of Quantum System Engineering, Chonbuk National University, 567 Baekje-daero, Deokjin-gu, Jeonju-si, Jeollabuk-do, 54896, Republic of Korea

[2]Department of Applied Plasma Engineering, Chonbuk National University, 567 Baekje-daero, Deokjin-gu, Jeonju-si, Jeollabuk-do, 54896, Republic of Korea

*email: symoon@jbnu.ac.kr

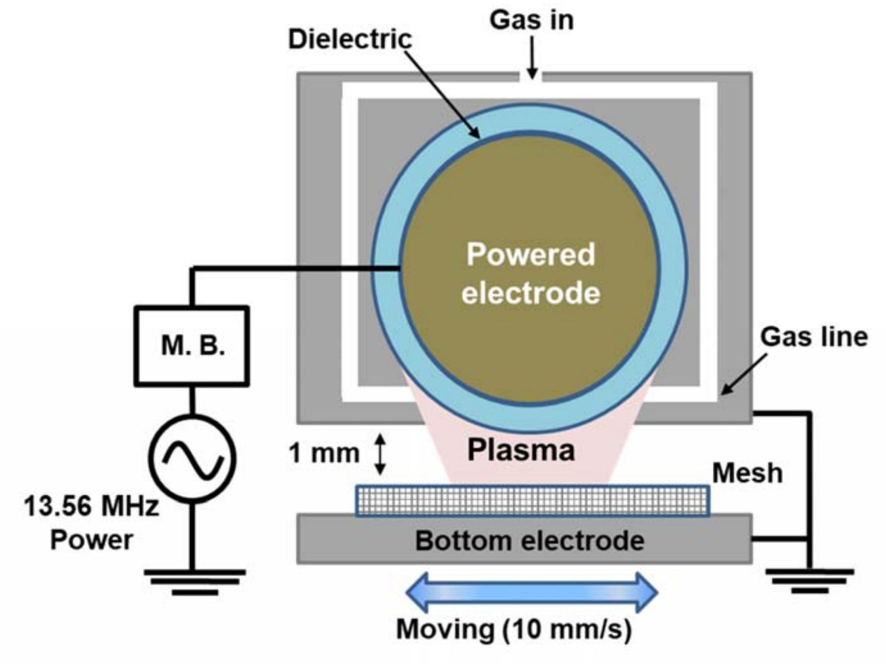

Figure S1. The schematic illustration of experimental setup: 13.56 MHz power feed through a matching box (M.B.) and He/CH_4/C_4F_8 gases are supplied and controlled their total flow and mixing ratio by mass flow controllers. An aluminum rod connected to the power supply is covered by a dielectric tube and both the outer body and sample plate were electrically grounded.

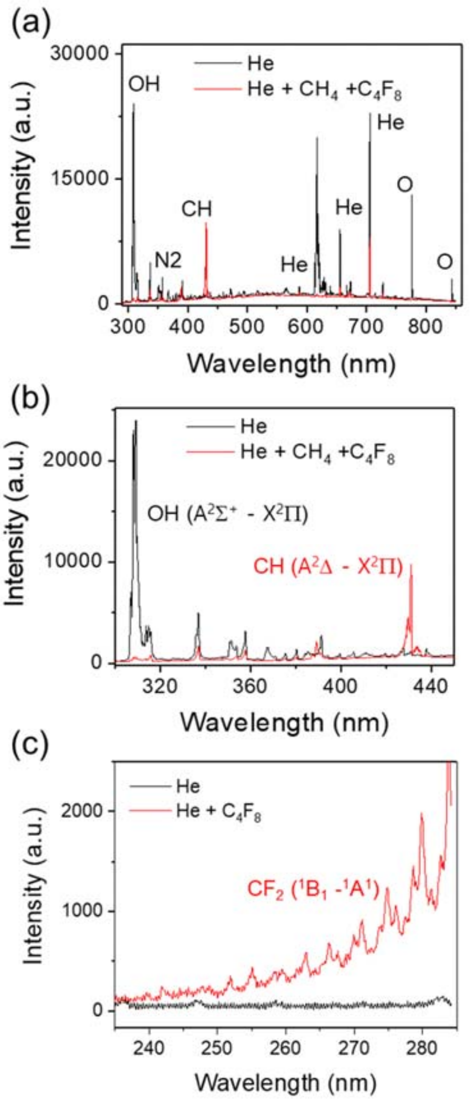

Figure S2. (a) Full visible emission spectra (b) and near ultra-violet emission spectra from pure helium and CH_4 and C_4F_8 added to helium plasma. (c) Ultra-violet emission spectra from pure helium and C_4F_8 added to helium plasma. In the pure helium plasma case, the strong OH, oxygen atomic lines and some nitrogen molecular emissions are observed because of open air processing. However, by adding CH_4 and/or C_4F_8, CH (A-X) and/or CF_2 (B-A) molecular emission bands are observed.

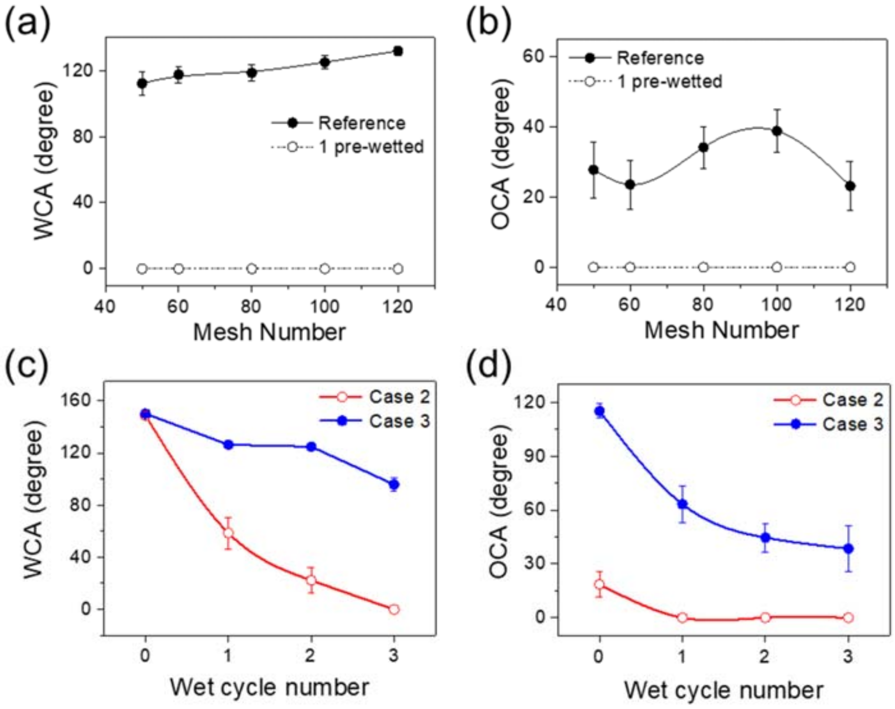

Figure S3. (a) WCA and (b) OCA of reference stainless steel membranes for various mesh numbers (dots) and contact angle values after a ‘wet cycle’, i.e. an immersion in a water bath for 2 minutes (circles). (c) WCA and (d) OCA on single-side plasma treated membranes with case 2 and case 3 for various wet cycles numbers (performed on a membrane with a mesh number of 120).

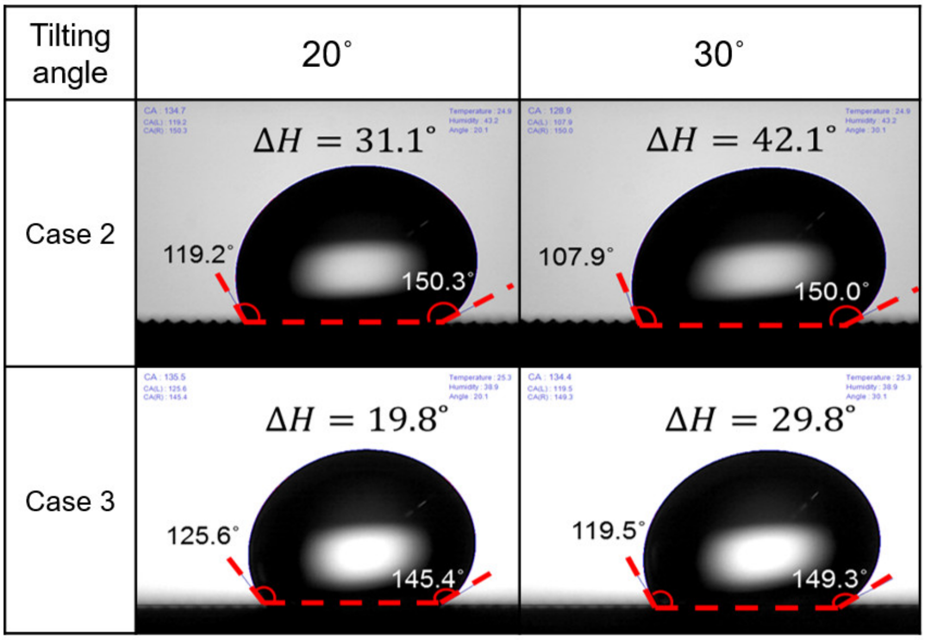

Figure S4. Advancing and receding angles measurements of plasma treated membrane surfaces with case 2 and case 3 using the tilting method. The hysteresis values defined as the difference between advancing and receding angles are estimated at 20° and 30° of tilting angle.

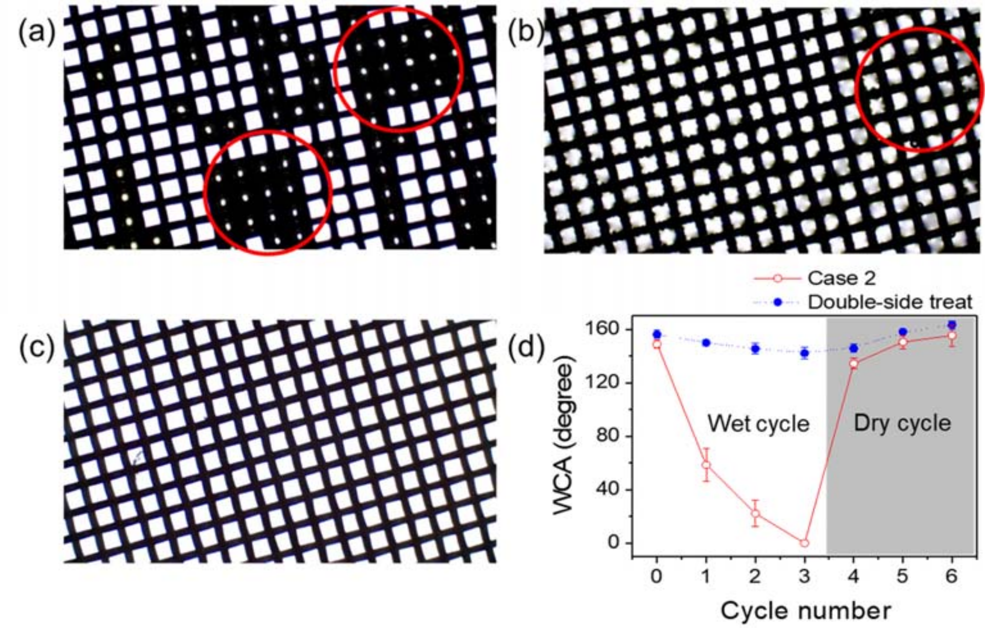

Figure S5. Optical micrographs of (a) reference mesh, (b) one-side plasma treated mesh with case 2 and (c) double-sided plasma treated mesh with case 2 and 3 after dipping in water for 2 minutes (wet cycle test). Water residues (red circles) trapped in the membrane pores in reference mesh and case 2 are found, which leads to the decrease of the WCA. (d) The WCA of case 2 (red empty circle) was restored to its initial value by dry cycle test (gray colored area) due to the dry of trapped water residue by wet cycle. In the case of the double side treated mesh, the changes of WCA (blue solid circle) were not negligible whatever the wet or dry cycle.

Movie clip. Oil-water separation test performed using a selectively functionalized mesh membrane with case 2 (front side) and case 3 (back side) at 3rd wet-cycle test (double speed).
